# Movement-related increases in subthalamic activity optimize locomotion

**DOI:** 10.1016/j.celrep.2025.116894

**Published:** 2025-12-29

**Authors:** Joshua W. Callahan, Juan Carlos Morales, Jeremy F. Atherton, Dorothy Wang, Selena Kostic, Mark D. Bevan

Several elements were missing from the original paper.

The additional affiliation “Aligning Science Across Parkinson’s (ASAP) Collaborative Research Network, Chevy Chase, MD 20815, USA” should have been specified for each author.

The following ORCID links should have been cited: Joshua W. Callahan (https://orcid.org/0000-0001-7567-5676), Jeremy F. Atherton (https://orcid.org/0000-0002-4144-0657), and Mark D. Bevan (https://orcid.org/0000-0001-9759-0163).

The statement “The data used to generate figures and tables are available from https://zenodo.org/records/14420608” should have been included in the data and code availability section of the manuscript.

Three entries were missing from the software and algorithms section of the key resources table. These additions are as follows:

**Table:** 

Clampex 11	Molecular Devices	http://www.moleculardevices.com/products/software/pclamp.html; RRID: SCR_011323
LinLab	Scientifica	https://www.scientifica.uk.com/products/scientifica-linlab-2
SpinView	Teledyne Flir	https://softwareservices.flir.com/spinnaker/latest/_spin_view_guide.html

The authors regret any inconvenience that may have been caused by these omissions.

